# Laboratory Hints

**Published:** 1906-06

**Authors:** J. F. Steele

**Affiliations:** Eagle Grove, Iowa.


### LABORATORY HINTS.

J. F. Steele, D. D. S., Eagle Grove, Iowa.

I frequently have patients whose plates become loose and need refitting but they do not like the idea of giving up the old teeth, as they have become used to the expression of the old set and fear that they are taking some risk in having them reset. The work may be easily done and the same expression retained by the following method.

Take impression and make plaster cast as usual. Put old plate on cast and paste firmly with Fowler's Sticky wax. Now fasten cast in articulator in the usual way for a bite. After plaster is set take some modelling compound, soften and fasten to the other half of articulator. Make smooth on top surface, close it up, and force the tooth into it. Now remove the teeth as soon as cool and mount them on a new cast and proceed as you would with any ordinary case, always paying attention, of course, to all the little important details that must never be neglected in any case.

